# Practice of hemodynamic monitoring and management in German, Austrian, and Swiss intensive care units: the multicenter cross-sectional ICU-CardioMan Study

**DOI:** 10.1186/s13613-016-0148-2

**Published:** 2016-05-31

**Authors:** Sandra Funcke, Michael Sander, Matthias S. Goepfert, Heinrich Groesdonk, Matthias Heringlake, Jan Hirsch, Stefan Kluge, Claus Krenn, Marco Maggiorini, Patrick Meybohm, Cornelie Salzwedel, Bernd Saugel, Gudrun Wagenpfeil, Stefan Wagenpfeil, Daniel A. Reuter, for the ICU-CardioMan Investigators

**Affiliations:** 1 Department of Anaesthesiology, Centre of Anaesthesiology and Intensive Care Medicine, University Medical Centre Hamburg-Eppendorf, Martinistrasse 52, 20246 Hamburg, Germany; 2 Department of Anaesthesiology and Intensive Care Medicine, UKGM University Hospital Gießen, Justus-Liebig-University Giessen, Rudolf-Buchheim-Strasse 7, 35392 Giessen, Germany; 3 Department of Anaesthesiology, Critical Care Medicine and Pain Medicine, University Hospital of Homburg/Saar, Kirrberger Strasse 100, 66421 Homburg, Germany; 4 Department of Anaesthesiology and Intensive Care Medicine, University of Luebeck, Ratzeburger Allee 160, 23538 Luebeck, Germany; 5 Department of Anaesthesia, Intensive Care, Emergency and Pain Medicine, Hospital Mechernich, St.-Elisabeth-Strasse 2-6, 53894 Mechernich, Germany; 6 Department of Anaesthesiology, University of Vienna, Währinger Gürtel 18-20, 1090 Vienna, Austria; 7 Department of Intensive Care Medicine, University of Zurich, Rämistrasse 100, 8091 Zurich, Switzerland; 8 Department of Anaesthesiology and Intensive Care Medicine, University of Frankfurt, Theodor-Stern-Kai 7, 60590 Frankfurt, Germany; 9 Department of Clinical Medicine, Saarland University, Campus Homburg, Kirrberger Strasse 100, 66421 Homburg, Germany; 10 Institute for Medical Biometry, Epidemiology and Medical Informatics, Saarland University, Campus Homburg, Kirrberger Strasse 100, 66421 Homburg, Germany

**Keywords:** Hemodynamic management, Treatment protocol, Guidelines, Echocardiography, Thermodilution, Pulse contour analysis, Cardiac output, Pulse pressure variation, Stroke volume variation

## Abstract

**Background:**

Hemodynamic instability is frequent and outcome-relevant in critical illness. The understanding of complex hemodynamic disturbances and their monitoring and management plays an important role in treatment of intensive care patients. An increasing number of treatment recommendations and guidelines in intensive care medicine emphasize hemodynamic goals, which go beyond the measurement of blood pressures. Yet, it is not known to which extent the infrastructural prerequisites for extended hemodynamic monitoring are given in intensive care units (ICUs) and how hemodynamic management is performed in clinical practice. Further, it is still unclear which factors trigger the use of extended hemodynamic monitoring.

**Methods:**

In this multicenter, 1-day (November 7, 2013, and the preceding 24 h) cross-sectional study, we retrieved data on patient monitoring from ICUs in Germany, Austria, and Switzerland by means of a web-based case report form. One hundred and sixty-one intensive care units contributed detailed information on availability of hemodynamic monitoring. In addition, detailed information on hemodynamic monitoring of 1789 patients that were treated on due date was collected, and independent factors triggering the use of extended hemodynamic monitoring were identified by multivariate analysis.

**Results:**

Besides basic monitoring with electrocardiography (ECG), pulse oximetry, and blood pressure monitoring, the majority of patients received invasive arterial (77.9 %) and central venous catheterization (55.2 %). All over, additional extended hemodynamic monitoring for assessment of cardiac output was only performed in 12.3 % of patients, while echocardiographic examination was used in only 1.9 %. The strongest independent predictors for the use of extended hemodynamic monitoring of any kind were mechanical ventilation, the need for catecholamine therapy, and treatment backed by protocols. In 71.6 % of patients in whom extended hemodynamic monitoring was added during the study period, this extension led to changes in treatment.

**Conclusions:**

Extended hemodynamic monitoring, which goes beyond the measurement of blood pressures, to date plays a minor role in the surveillance of critically ill patients in German, Austrian, and Swiss ICUs. This includes also consensus-based recommended diagnostic and monitoring applications, such as echocardiography and cardiac output monitoring. Mechanical ventilation, the use of catecholamines, and treatment backed by protocol could be identified as factors independently associated with higher use of extended hemodynamic monitoring.

**Electronic supplementary material:**

The online version of this article (doi:10.1186/s13613-016-0148-2) contains supplementary material, which is available to authorized users.

## Background

Cardiocirculatory dysfunction with subsequent hemodynamic instability is a frequent and crucial symptom found in many medical conditions requiring intensive care therapy. Hemodynamic instability diminishes oxygen supply to the end organs and is associated with an increased mortality rate [1]. Thus, hemodynamic management represents a cornerstone of intensive care therapy and has therefore been addressed by an increasing number of guidelines and recommendations [1–4]. Pulmonary artery catheterization, various forms of indicator dilution, arterial pulse wave analysis, and in particular the increasing availability of ultrasound technologies nowadays allow for closely monitor cardiocirculatory function. This comprises—besides blood pressures—the measurement of blood flow, contractile function of the heart, and metabolic parameters giving information about oxygen demand and consumption. Though recently challenged with regard to an approach originally promoted by Rivers et al. [5] for patients with severe sepsis and septic shock, goal-directed strategies of hemodynamic management based on parameters of extended hemodynamic and metabolic monitoring are increasingly recommended in different national and international guidelines, in particular for peri- and postoperative care [6–10]. However, how hemodynamic monitoring is actually practiced in intensive care units (ICU) is only scarcely described. This comprises the question whether the tools and the infrastructural resources, which are necessary for the routine use of extended hemodynamic monitoring, are available in all institutions. Further, it remains unclear which factors trigger the use of extended hemodynamic monitoring in practice. So far, it is also not known whether and what kind of therapeutic consequences arise from the implementation of extended hemodynamic monitoring.

The purpose of this cross-sectional study was to characterize how cardiovascular function is monitored in critically ill patients in three European countries (Germany, Austria, and Switzerland). We further aimed to identify patient- or healthcare-related factors that trigger the use of advanced hemodynamic monitoring.

## Methods

### Study design

This cross-sectional study was initiated by the scientific working group “Intensive Care Medicine” of the German Society of Anaesthesiology and Intensive Care Medicine (DGAI) and was endorsed by the Austrian Society for Anaesthesiology, Resuscitation, and Intensive Care Medicine (OGARI), the Swiss Society of Intensive Care Medicine (SGI), the German Society of Trauma Surgery (DGU), the German Interdisciplinary Association of Intensive Care and Emergency Medicine (DIVI), the Federation of Austrian Societies of Intensive Care Medicine (FASIM), and the European Society of Intensive Care Medicine (ESICM).

The ethics committees of the University Homburg/Saar, Germany, reviewed and approved the study protocol (Ethik-Kommission der Ärztekammer des Saarlandes mit Kenn-Nr. Ha07/13). The need for written informed consent was waived since the research involved no risk to the patients, and patient data were anonymized before transmission to the database. We directly contacted members of the aforementioned societies and associations via email or via the internet presentations of the societies and invited them to electronically register their ICUs for participation in this study. Registered units were then provided with the detailed study protocol and were instructed how to approach approval from their local ethics committee. Registration for the study was only completed with the written approval of the respective local ethics committee.

### Data collection

Data collection was performed via a web-based case report form (CRF). This CRF consisted of two parts. Part one gathered general information about the hospital, the respective ICU, the monitoring equipment available in that ICU, and implemented treatment strategies (treatment algorithms, standard operating procedures). Part two then enquired specific information about the hemodynamic monitoring and hemodynamic management strategy used in each individual patient treated in the respective unit on November 7, 2013, and the preceding 24 h.

### Endpoints

We performed descriptive analyses of the participating medical centers and their ICUs. Then, we evaluated the availability of technical equipment and related monitoring options. We further investigated whether standardized treatment plans (i.e., algorithms or standard operating procedures) were implemented for hemodynamic management. Next, we analyzed whether the availability of the different monitoring options was associated with hospital size, academic status of the hospital, the leading medical discipline in the ICU, or the fact that standardized treatment plans were implemented.

Furthermore, we characterized the patients that participated with their data in this study. We analyzed the reasons for admission to the ICU, their leading diagnosis, and the severity of illness quantified by the simplified acute physiology score (SAPS) of the included patients in each participating ICU. We investigated how many patients required catecholamines or vasopressors. In order to determine how many patients qualified for the use of functional parameters of preload, such as pulse pressure or stroke volume variation, we characterized who was on mechanical ventilation and which kind of cardiac rhythm was present in those patients.

After descriptive analyses, we investigated by a multivariate regression analysis which factors were independently associated with the choice of different monitoring modalities. These factors comprised infrastructural (hospital size, academic affiliation, leading medical discipline, implemented treatment protocols) or patient-associated (leading diagnosis, severity of illness, mechanical ventilation, need of catecholamine or vasopressor support) aspects.

Finally, we analyzed whether an escalation of cardiovascular monitoring within the last 24 h influenced treatment strategies in the included patients.

### Statistical analyses

Data analysis was performed using IBM SPSS Statistics for Windows, version 22.0 (IBM Corp., Armonk, NY, USA), and StatsDirect, version 2.7.9 (StatsDirect LTD, Cheshire, UK). All tests were conducted two-sided in an explorative manner on a 5 % significance level. For descriptive statistical analysis, we calculated absolute and relative frequencies (in percentage) to describe categorical data and mean ± standard deviation and median as well as range for continuous data. To identify factors independently associated with the use of extended hemodynamic monitoring modalities, we performed multiple logistic regression models using a stepwise forward and backward variable selection approach. Based on these statistical models, odds ratio estimates (OR) were calculated with 95 % confidence intervals (CI). In the multiple regression analysis, we included all factors showing a *p* value of ≤0.05 in univariate analyses. For variable selection in multiple regression analysis, we considered a *p* value of 0.10 to indicate statistical significance.

## Results

One hundred and sixty-one out of 165 initially registered ICUs contributed data regarding their infrastructure, their manning, the availability of monitoring equipment and monitoring standards, implemented treatment algorithms and standard operating procedures, as well as data from 1798 patients to this study. After removal of nine incomplete questionnaires, data from 1789 patients remained for analysis.

### Participating centers and their monitoring resources

**Fig. 1:**
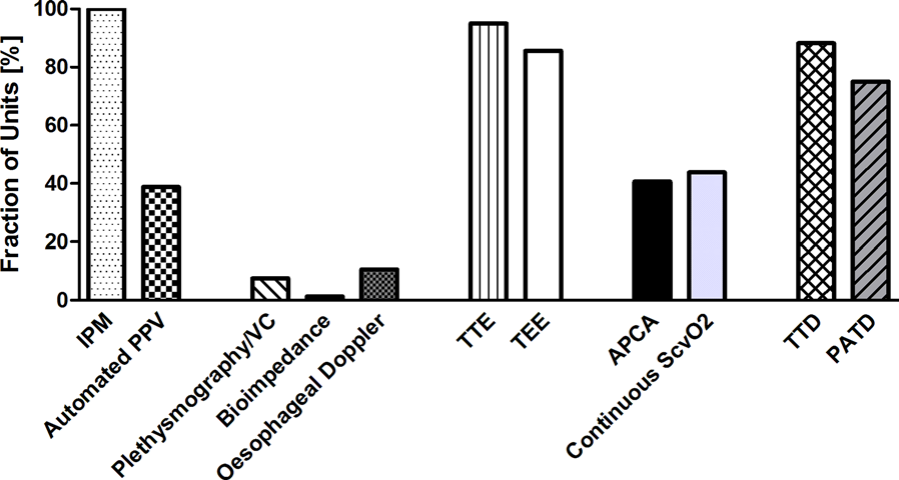
Availability of extended monitoring modalities. This figure depicts the different extended monitoring modalities and the percentages of units which have those available at the bedside (*IPM* invasive pressure monitoring, *PPV* pulse pressure variation, *VC* volume clamp, *TTE* transthoracic echocardiography, *TEE* transesophageal echocardiography, *APCA* autocalibrated pulse contour analysis, *ScvO2* central venous oxygen saturation, *TTD* transpulmonary thermodilution, *PATD* pulmonary artery thermodilution)

Characteristics of the participating centers and their ICUs are given in Table 1. 60.0 % of the participating units were at university hospitals. Figure 1 illustrates the all-over availability of extended hemodynamic monitoring, i.e., monitoring entities going beyond basic monitoring with electrocardiography (ECG), intermittent noninvasive blood pressure measurement, and pulse oximetry. Echocardiography (transthoracic or transesophageal) was available in 95.0 % and 85.7 %, and monitors using thermodilution (transpulmonary and pulmonary arterial) were available in 88.2 and 75.0 % of the participating units. In Table 2, those data are stratified according to the unit-leading medical discipline. A stratification of those availabilities of extended hemodynamic monitoring according to the size of the hospital and if the unit was part of a university hospital is given in table a1 (Additional file 1: Table a1).

**Table 1:** Characterization of the 161 participating centers and their intensive care units

	*n*
*Hospital size*	
≤500 beds	26 (16 %)
501–1000 beds	48 (30 %)
>1000 beds	87 (54 %)
University hospital	97 (60 %)
Non-university Hospital	64 (40 %)
*Type of ward*	
ICU	114 (70.8 %)
IMC	12 (7.5 %)
Mixed	35 (21.7 %)
*Infrastructure of ward*	
Number of beds/ward	14.7 ± 8.4 Median: 12 (4–64)
Number beds/physician	5.5 ± 2.6 Median: 5 (1.5–16)
Number of beds/nurse	2.4 ± 0.7 Median: 2.3 (0.8–7.2)
Algorithms implemented	118 (73.3 %)
*Leading disciplines*	
Anaesthesia	76 (47.2 %)
Medical	14 (8.7 %)
Neurology	4 (2.5 %)
Surgery	12 (7.5 %)
Interdisciplinary	28 (17.4 %)
Cardiac surgery	18 (11.2 %)
Others	9 (5.6 %)

**Table 2:** Available monitoring modalities stratified to unit-leading medical discipline

	Anaesthesia	Surgery	Cardiac surgery	Medical	Neurology	Interdisciplinary	Others
	*n* = 76 (%)	*n* = 12 (%)	*n* = 17 (%)	*n* = 14 (%)	*n* = 4 (%)	*n* = 28 (%)	*n* = 9 (%)
*Invasive pressure monitoring*							
Invasive pressure monitoring	100	100	100	100	100	100	100
Automated PPV	39.5	41.7	41.2	42.9	50	35.7	22.2
*Semi*-*invasive extended monitoring*							
Finger plethysmography	7.9	8.3	5.9	7.1	25	7.1	0
Bioimpedance	1.3	0	0	0	0	3.6	0
Esophageal Doppler	10.5	8.3	5.9	7.1	0	17.9	11.1
*Echocardiography*							
Transthoracic	96.1	91.7	100	100	75	92.9	88.9
Transesophageal	90.8	58.3	94.1	100	50	75	88.9
*Semi*-*invasive extended monitoring*							
Autocalibrated pulse contour analysis	51.3	25	43.8	35.7	0	21.4	55.6
Continuous ScvO2	28.9	33.3	37.5	35.7	0	25	55.6
*Invasive extended hemodynamic monitoring*							
Transpulmonary thermodilution	96.1	75	81.3	92.9	75	82.1	88.9
Transpulmonary lithium dilution	6.6	0	0	7.1	0	3.6	0
Pulmonary artery thermodilution	80.3	58.3	100	92.9	25	53.6	77.8

### Hemodynamic treatment standards

Figure a1 (Additional file 2: Figure a1) shows the proportion of units which had implemented treatment protocols relevant for hemodynamic management. Protocols for treatment of sepsis were implemented in 70 % of all units, as well as “other guidelines” such as institutional standard operating procedures. Table 3 gives detailed information, which treatment protocols were implemented stratified according to the unit-leading disciplines. In table a2 (Additional file 3: Table a2), this information is stratified according to the size and kind of hospital.

**Table 3:** Implemented hemodynamic treatment protocols stratified to unit-leading medical discipline

	Anaesthesia *n* = 76 (%)	Surgery *n* = 12 (%)	Cardiac surgery *n* = 17 (%)	Medical *n* = 14 (%)	Neurology *n* = 4 (%)	Interdisciplinary *n* = 28 (%)	Others *n* = 9 (%)
Septic shock	67.1	83.3	64.7	64.3	75	82.1	55.6
Cardiac surgery	25	33.3	76.5	7.1	0	14.3	44.4
Neurosurgery	35.5	58.3	5.9	0	50	17.9	33.3
Trauma	56.6	75	5.9	0	25	42.9	44.4
Myocardial infarction	32.9	25	35.3	57.1	25	39.3	11.1
Others	13.2	25	11.8	28.6	25	21.4	11.1

### Patient data

All together, we analyzed data of 1789 patients. Information on the reason for ICU admission, if it was a scheduled admission, and information, to which kind of unit patients were admitted, are given in table a3 (see Additional file 4: Table a3). Accordingly, 50.0 % of admissions were postsurgery and 45.8 % were medical emergencies. Further, data on intensive care scoring as well as information, if a hemodynamic treatment plan was used in the respective patient, are given. 48.9 % of the patients were mechanically ventilated, 39.2 % received catecholamines, and 58 % were treated based on a guideline or a treatment protocol.

We also retrieved detailed information on the main diagnosis relevant for the treatment in the ICU. According to those main diagnoses, patients were stratified to four clusters: Cluster “surgery” included all patients with a surgical (except cardiac surgical) procedure immediately prior to admittance to the ICU (*n* = 690); the cluster “cardiac surgery” comprised all patients following cardiac surgery procedures (*n* = 451); whereas in the cluster “medical” all patients were included with a primarily medical diagnosis, in the cluster “neuro,” all patients were included with a primarily neurological diagnosis, which was not associated with a surgical procedure.

Cardiac surgery was analyzed separately from surgical patients, since cardiovascular treatment in the ICU in this group is in particular determined by the consequences of intraoperative myocardial ischemia and reperfusion and the use of extracorporeal circulation.

### Practice of hemodynamic monitoring

Basic hemodynamic monitoring: In nearly all patients, basic hemodynamic monitoring, i.e., continuous ECG (*n* = 1753; 97.7 %), pulse oximetry (*n* = 1767; 98.4 %), and blood pressure monitoring, was performed. The latter was performed either noninvasively with a blood pressure cuff (*n* = 581; 32.5 %) or invasively (*n* = 1393; 77.9 %).

**Fig. 2:**
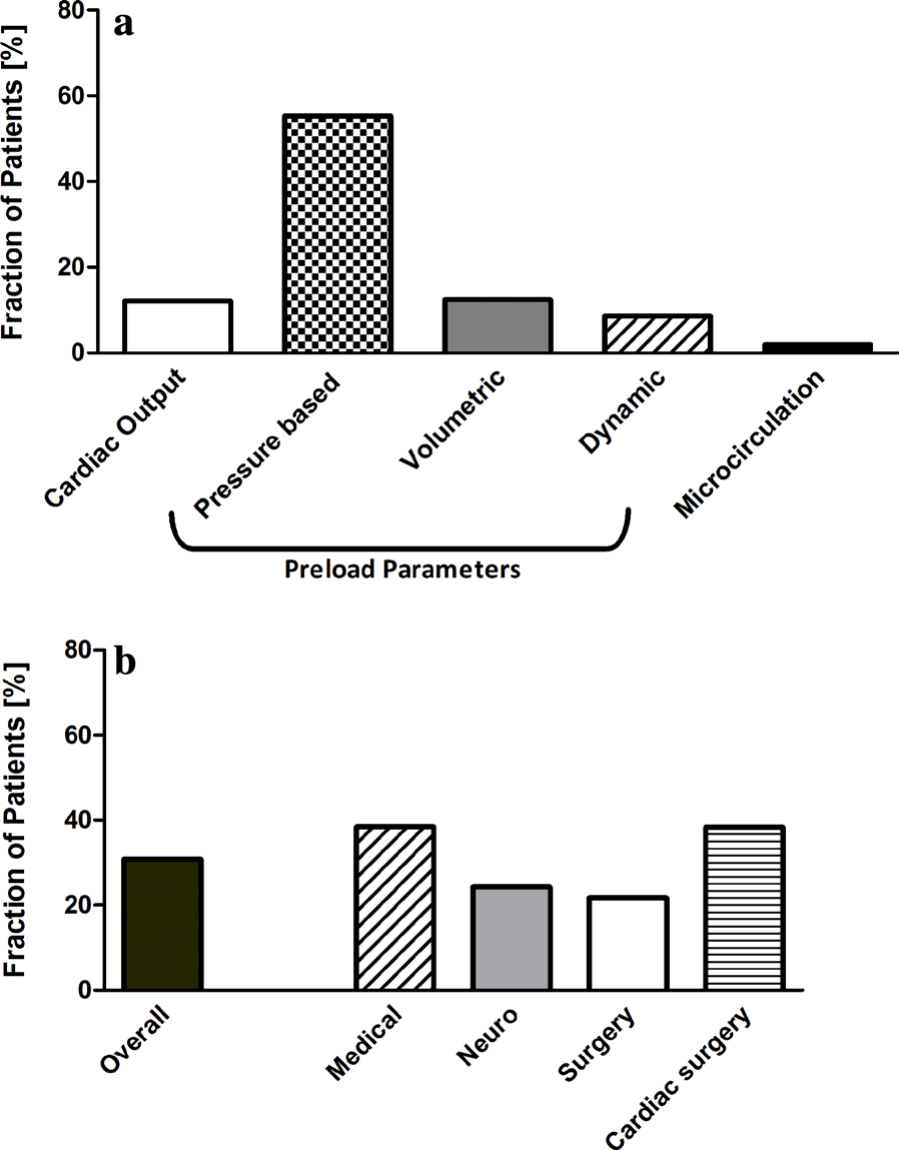
Use of extended hemodynamic monitoring. **a** The degree of use of measuring extended hemodynamic monitoring divided into the three subdomains: cardiac output, preload parameters, and microcirculation. **b** The degree of extended hemodynamic monitoring in general clustered by primary diagnosis

Extended hemodynamic monitoring: Fig. 2a depicts which kind of extended hemodynamic monitoring was actually used in the ICUs. In Fig. 2b, this information is stratified per primary diagnosis. Table 4 then lists which forms of extended hemodynamic monitoring were performed in the studied patients, again stratified to clusters of primary diagnosis.

**Table 4:** Implemented extended monitoring clustered per primary diagnosis and need for treatment with vasoactive agents or mechanical ventilation

Implemented monitoring	Primary diagnosis				Vasoactive agents	Mechanical ventilation
	Medical	Neuro	Surgery	Cardiac surgery		
	*n* = 504 (%)	*n* = 144 (%)	*n* = 690 (%)	*n* = 451 (%)	*n* = 702 (%)	*n* = 874 (%)
Basic	96.2	98.6	97.8	99.1	98.9	99.3
Basic plus central venous and/or arterial line	85.3	79.2	85.2	94.2	98.6	96.1
Extended	38.5	24.3	21.7	38.4	45.3	39.6
Cardiac output	12.3	4.2	10.3	17.8	24.2	20.8
Pressure-based preload parameters	44.4	37.5	51.6	79.2	74.4	69.5
Volumetric preload parameters	17.5	4.2	9.3	13.1	21.3	17.3
Dynamic preload parameters	9.3	5.5	9.7	7.3	16.5	14.9
Microcirculation	3.2	0.7	0.3	3.6	3.8	3.2

**Fig. 3:**
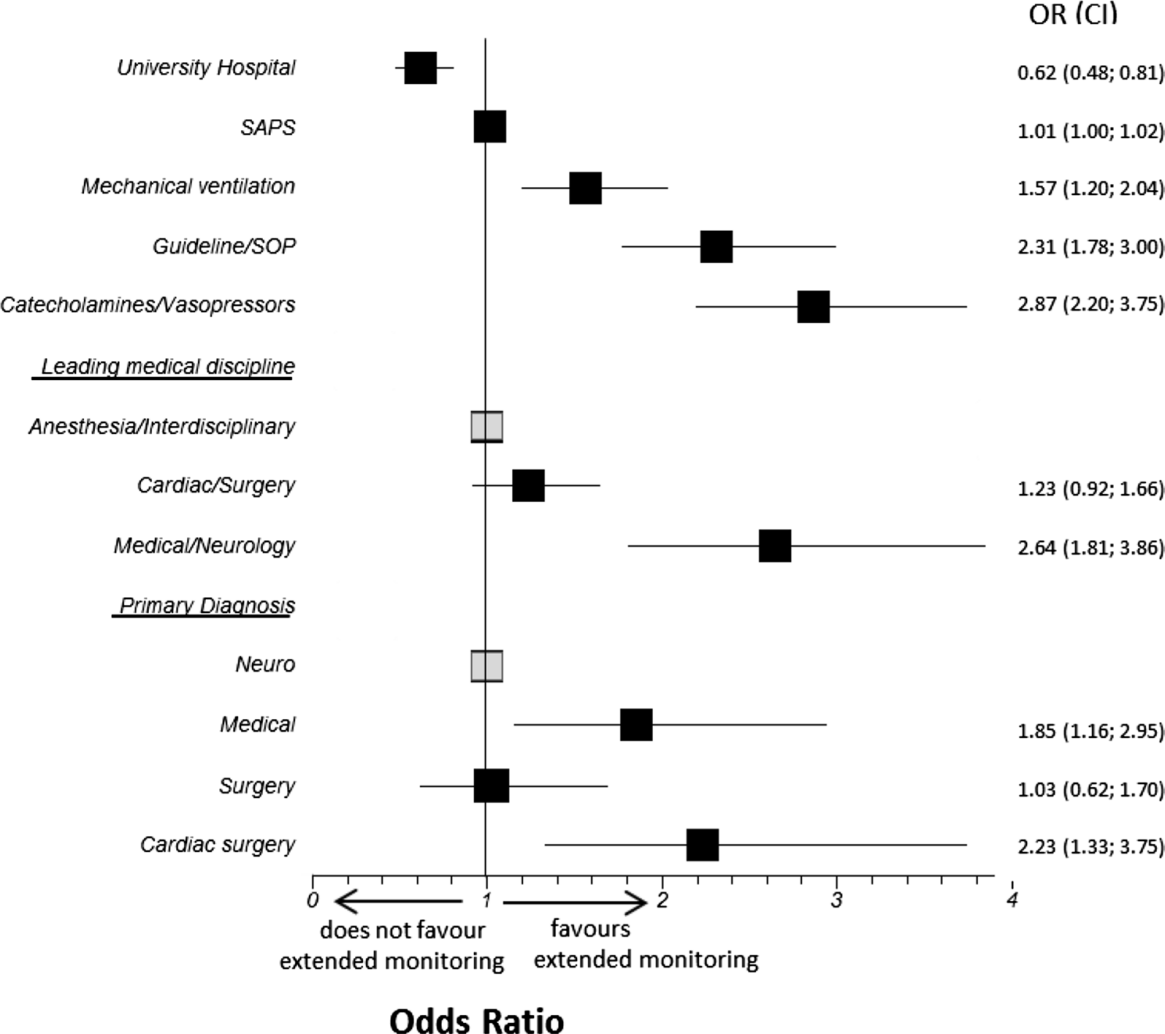
Independent factors to the use of extended hemodynamic monitoring. This figure plots the strengths of associations between specific infrastructural and patient characteristics as independent factors to the use of extended hemodynamic monitoring. Items marked in gray served as the respective reference categories

Figure 3 plots the associations and their strengths between specific infrastructural and patient characteristics as factors independently associated with to the use of extended hemodynamic monitoring, which we retrieved by multivariate regression analysis. The use of catecholamines (OR 2.87 (CI 2.2–3.75)], treatment backed on protocols [OR 2.31 (CI 1.78–3.0)], and mechanical ventilation [OR 1.57 (CI 1.20–2.04)] were the strongest factors positively associated with the use of extended hemodynamic monitoring. Further positive predictors were a primarily medical [OR 1.85 (CI 1.16–2.95)] or cardiac surgical [OR 2.23 (CI 1.33–3.75)] diagnosis, and the leading medical discipline being medicine or neurology [OR 2.64 (CI 1.81–3.86)].

### Changes in hemodynamic monitoring and resulting therapeutic consequences

The addition of one or more hemodynamic monitoring tools during the last 24 h was reported in 116 patients. This was in 54 (46.6 %) patients the implementation of an arterial catheter, in 35 (30.2 %) patients the implementation of a central venous catheter, in 34 (29.3 %) patients the performance of one or multiple echocardiographic examinations (either transthoracic or transesophageal), in 22 (19.0 %) patients the implementation of transpulmonary thermodilution, in 8 (6.9 %) patients the insertion of a pulmonary artery catheter, in 4 (3.5 %) patients the implementation of autocalibrated pulse contour analysis, and in 22 (19.0 %) patients the addition of other monitoring techniques. In 83 of the 116 patients, direct therapeutic consequences (71.6 %) because of this monitoring escalation were reported.

## Discussion

The results of this cross-sectional study reflect how hemodynamic monitoring is performed in reality in ICUs in the three European countries Germany, Austria, and Switzerland. In fact, all patients were monitored with basic hemodynamic monitoring consisting of ECG, blood pressure measurements, and pulse oximetry. Also the majority of patients received invasive arterial and central venous catheterization. Extended hemodynamic monitoring, and in particular monitoring of cardiac output, although widely available, was performed all over in less than 15 %. Mechanical ventilation, treatment backed by protocols, and the need for catecholamine therapy were the factors independently associated with the use of advanced hemodynamic monitoring. This was also validated in subgroup analyses of patients treated by mechanical ventilation or vasoactive agents. The most frequently used method for preload monitoring was the assessment of filling pressures. The use of volumetric or dynamic parameters of preload was rare. In 71.6 % of patients, in whom extended hemodynamic monitoring was added during the study period, this addition of monitoring led to direct changes in treatment. The vast majority of ICUs reported that protocols for hemodynamic treatment were available, and in nearly 60 % of patients, hemodynamic management was reported to be performed according to an implemented treatment protocol. In particular, treatment protocols for patients with sepsis were established. Whether quality of cardiovascular treatment was influenced by the use of specific treatment protocols cannot be assessed on the basis of the present data.

The acknowledgement that hemodynamic instability influences morbidity and mortality of critically ill patients has led to the development of various techniques of advanced hemodynamic monitoring and cardiovascular imaging. Further, many technical approaches have been taken to measure even complex physiological signals with low or even no invasiveness in order to reduce the monitoring-associated risks [11]. Finally, guidelines, treatment protocols, and consensus-based recommendations for several major diagnoses relevant for intensive care treatment have been developed in order to secure the transfer of best practice of hemodynamic management based on pathophysiological rationale and scientific evidence. But how this has influenced practice of hemodynamic monitoring and management in reality has only scarcely been investigated. Data regarding availability of monitoring equipment based on smaller surveys were published earlier from Switzerland including 55 medical and surgical ICUs, from Germany in 55 cardiac surgery ICUs, as well as from Italy again in 71 cardiac surgery centers [12–14]. Strength of our data set is that it represents data from a broad spectrum of ICUs of different sizes and a balanced proportion of ICUs at both university hospitals and non-university hospitals. This allows a comparison between different disciplines and institutions. Furthermore, we collected not only data on general availability of monitoring equipment but also real patient data, showing which monitoring was actually used. This, in combination with a high number of patients included, allowed further identification of factors that independently triggered the use of extended hemodynamic monitoring.

Our data show that technical requirements for basic hemodynamic monitoring, i.e., ECG, noninvasive blood pressure monitoring, and pulse oximetry, were given on all units. Noninvasive, extended hemodynamic monitoring (finger plethysmography waveform analysis, continuous blood pressure monitoring by volume clamp methods, or esophageal Doppler) was only available in very few of the studied ICUs, which is in line with earlier data from Switzerland [12]. Of note, there was obviously no relevant difference between the unit-leading disciplines regarding availability of these noninvasive technologies. Whether this low availability was caused by missing confidence in monitoring accuracy or by other reasons (for example economic) was beyond the scope of the present study.

In contrast, availability of echocardiography was much higher. Transthoracic echocardiography was available in more than 90 % of all units, with no major differences between different sizes of hospitals, between university hospitals and non-university hospitals, or between the different unit-leading disciplines. Obviously, today also transesophageal echocardiography is widely available (i.e., in more than 80 % of the units). This underlines the high acceptance and appreciation of this technology in intensive care medicine also outside cardiac surgery [13, 14]. But this high availability and the strong recommendations for its use [4, 15] are in contrast to the very low number of patients (34 out of 1790 patients, i.e., 1.9 %) that were actually reported to be investigated by echocardiography during the 24-h study period. Interestingly, Boulain et al. demonstrated comparable numbers specifically regarding the use of echocardiography for hemodynamic management of shock [16]. Further, echocardiography is only scarcely used in practice in Europe for guiding fluid therapy as Cecconi et al. recently showed [17].

We defined semi-invasive extended hemodynamic monitoring as monitoring modalities which use either peripheral arterial or central venous vascular access for further analyses besides pressure monitoring (i.e., autocalibrated arterial pulse contour analysis) or continuous central venous oximetry. It is of interest that, although nearly all of the investigated patients had arterial and central venous catheters in place, these monitoring modalities were only available in the minority of units.

Invasive extended hemodynamic monitoring based on thermodilution was widely available. Besides in cardiac surgery, which seems to remain a leading domain of the pulmonary artery catheter, the most frequent technology was transpulmonary thermodilution. However, the actual all-over use of both monitoring modalities is comparably low, as reflected in table a1 (Additional file 1: Table a1). This low use is supported also by the recent data from French ICUs, which points out the divergence between subjective perception of higher use reflected in the results of surveys among ICU physicians and the objective assessment based on patient data [12–14, 16].

Adequate monitoring and management of cardiac preload, and in particular the inaccuracy of the cardiac filling pressures CVP and PAOP as the rationale for guiding fluid therapy, have recently led many scientific discussions. Although filling pressures can offer additional and relevant physiological information, current consensus statements based on recently published data recommend not to manage fluid therapy primarily by filling pressures [1, 18]. From that perspective, it is interesting that still filling pressures were the main tool for the assessment of preload, whereas volumetric or functional parameters of preload played only a minimal role. This is again in line with the French findings of Boulaint et al. in patients with septic shock [16] and also with a recently published French study by Preau et al. investigating the use of static and dynamic hemodynamic parameters for predicting fluid responsiveness prior to volume expansion [19]. One explanation may be the frequently stressed limitations for the use of automated functional parameters of preload, i.e., the necessity of controlled mechanical ventilation and the absence of significant arrhythmias. This can be partly substantiated by our data. Although more than 80 % of all patients presented with a cardiac rhythm that allowed interpretation of those parameters (sinus rhythm, pacer rhythms), only 22.9 % of patients were on fully controlled mechanical ventilation. In contrast, in the group of hemodynamic unstable patients that received vasoactive agents, though the rate of arrhythmias was comparable, a higher ratio of 30.9 % fulfilled criteria for the assessment of functional preload parameters due to more frequent controlled mechanical ventilation. But even in this group, only about every second of these patients that fulfilled the criteria was monitored by dynamic preload parameters. Furthermore, thinking of the other two-thirds not fulfilling the criteria, it stresses the need for further development of functional parameters of preload that operate independently from the presence of ventilation mode. This is important as one can argue that those patients reflected presumably the group of patients with the highest need for differentiated cardiovascular management, because of the severity of their disease.

In particular, it is remarkable that the over-all fraction of patients monitored with cardiac output monitoring was as low as reported in 2003, i.e., more than 10 years ago, by Oldner et al. from 114 patients on Scandinavian ICUs [20]. Although in cardiac surgery patients and in patients receiving vasoactive agents, serving as a sign of hemodynamic instability, the proportion of patients in which cardiac output was monitored was now with 24 % slightly higher, the availability of novel and less invasive modalities for cardiac output monitoring has not extensively increased the use of this parameter so far. It is further remarkable that also the affiliation to a university hospital compared with a non-university hospital was no independent predictor for the use of extended hemodynamic monitoring.

The present study has limitations given by the fact that it was a point prevalence study, and should therefore be primarily understood as an initial assessment of status quo of hemodynamic monitoring and management. Further, inter- and intrapersonal reliability of the online CRF was not tested. Also the absence of more detailed background information on the specific hemodynamic protocols used in each individual institution, and the lacking information of clinical outcome limits further conclusions. Furthermore, the non-implementation of a standardized protocol cannot be automatically equated with the absence of knowledge on current guidelines or lower quality of patient care. However, treatment protocols were identified as one of the independent factors triggering the use of extended hemodynamic monitoring. Thus, they might moreover serve as an additional trigger for a closer hemodynamic evaluation in particular groups of patients. Here, further studies are desirable.

## Conclusion

The results of this study draw a representative picture, how hemodynamic monitoring and management is performed in intensive care medicine, and which factors independently favoured/refrained its use in three European countries. The study helped to reveal that extended hemodynamic monitoring, although available in most units, is applied only in a minor part for the surveillance of critically ill patients. Surprisingly, this included also consensus-based recommended diagnostic and monitoring applications, such as echocardiography and cardiac output monitoring. In the majority of patients, in which monitoring was extended, this escalation resulted in changes in treatment. The use of catecholamines, mechanical ventilation, and treatment on the basis of a protocol were independently associated with the use of extended hemodynamic monitoring. On the opposite, surgical patients were less exposed to extended hemodynamic monitoring compared with medical and cardiac surgery patients. The vast majority of ICUs reported that patients’ hemodynamic management was performed according to treatment protocols, in particular for sepsis.

## Additional files

**Additional file 1: Table a1.** Available monitoring modalities stratified according to hospital size and academic affiliation.

**Additional file 2: Figure a1.** Implemented treatment protocols Figure a1 illustrates, in how many percent of units the different treatment protocols, that are relevant for hemodynamic management, were implemented (MI = myocardial infarction).

**Additional file 3: Table a2.** Implemented haemodynamic treatment protocols stratified to size of hospital and academic affiliation.

**Additional file 4: Table a3.** Characteristics of included patients.

**Additional file 5: Table a4.** List of Co-Investigators.

## Abbreviations

AF: atrial fibrillation
APCA: autocalibrated pulse contour analysis
CI: confidence interval
CRF: case report form
CVP: central venous pressure
ECG: electrocardiography
ICU: intensive care unit
IMC: intermediate care unit
IPM: invasive pressure monitoring
OR: odds ratio
PAOP: pulmonary artery occlusion pressure
PATD: pulmonary artery thermodilution
PPV: pulse pressure variation
SAPS: simplified acute physiology score
S_c_VO_2_: central venous oxygen saturation
TEE/TTE: transesophageal echocardiography/transthoracic echocardiography
TISS: therapeutic intervention scoring system
TTD: transpulmonary thermodilution
VC: volume clamp
