# Erratum to: Practice of hemodynamic monitoring and management in German, Austrian, and Swiss intensive care units: the multicenter cross-sectional ICU-CardioMan Study

**DOI:** 10.1186/s13613-017-0297-y

**Published:** 2017-07-17

**Authors:** Sandra Funcke, Michael Sander, Matthias S. Goepfert, Heinrich Groesdonk, Matthias Heringlake, Jan Hirsch, Stefan Kluge, Claus Krenn, Marco Maggiorini, Patrick Meybohm, Cornelie Salzwedel, Bernd Saugel, Gudrun Wagenpfeil, Stefan Wagenpfeil, Daniel A. Reuter, for the ICU-CardioMan Investigators, Gerd Werner Albuszies, Rudolf Alexi, Andreas Bauer, Stefan Bergt, Ralph Berroth, Frank Bloos, Hendrick Bracht, Patrick Brass, Dirk Breukelmann, Andreas Brunauer, Matthias Burghardt, Michael Christ, Beatrix Dietrich, Christian Diwok, Christian Dohmen, Michael Dueck, Ingolf Eichler, Joerg Engel, Regula Erb, Florian Eyer, Andreas Faltlhauser, Florian Feld, Ann Kristin Fricke, Bernd Fröhmert, Wolfgang Funk, Harald Genzwuerker, Hans-Ulrich Giesen, Hans-Hinrich Grafelmann, Joachim Grosse, Marco Gruss, Christoph Gussone, Hendrik Haake, Markus Heim, Anke Heinemann, Michael Henrich, Holger Herff, Guido Hermanns, Klaus Hofmann-Kiefer, Andreas Hohn, Frauke Honig, Tobias Huebner, Tobias Hueppe, Nadine Huelpuesch, Judith Ibba, Ricarda Ibscher, Michael Irlbeck, Johannes Kalbhenn, Barbara Kapfer, Ines Kaufmann, Thomas Kirschning, Stephan F. Kloesel, Karin Kobusch, Matthias Kochanek, Alf Kozian, Oliver Krull, Thomas Kuhl, Simone Lindau, Ingo Lorenz, Stefan Maisch, Gernot Marx, Thomas Marx, Onnen Moerer, Benjamin Moser, Ulrike Mueller, Stefan Nicolas, Ludwig Ney, Andreas Niedeggen, Stefan Otto, Kevin Pilarczyk, Matthias Popp, Christian Putensen, Ralf Quabach, Maximilian Ragaller, Axel Ramminger, Stefan Rasche, Sebastian Rehberg, Marcus H. T. Reinges, Reimer Riessen, Knut-Age Roehrich, Rainer Roehrig, Jan P. Roesner, Heiner Ruschulte, Samir G. Sakka, Alexander Schellhaass, Jens-Christian Schewe, Ingo Schirotzek, Jörn V. Schley, Alexander Schmitt, Martin Schott, Patrick Schramm, Stefan Schroeder, Jan-Karl Schuette, Alexander Schuld, Konrad Schwarzkopf, Julia Seiler, Sixten Selleng, Philipp Simon, Kornel Skitek, Jens Soukup, Markus Stephan, Henning Stetefeld, Klaudiusz Suchodolski, Stefan Toennies, Georg Trummer, Michael Wattenberg, Stefan P. Wirtz, Hermann Wrigge, Christian Wunder, Cagatay Yildirim, Kai Zacharowski, Alexander Zarbock, York Zausig, Michael Zoller

**Affiliations:** 1 Department of Anaesthesiology, Centre of Anaesthesiology and Intensive Care Medicine, University Medical Centre Hamburg-Eppendorf, Martinistrasse 52, 20246 Hamburg, Germany; 2 Department of Anaesthesiology and Intensive Care Medicine, UKGM University Hospital Gießen, Justus-Liebig-University Giessen, Rudolf-Buchheim-Strasse 7, 35392 Giessen, Germany; 3 Department of Anaesthesiology, Critical Care Medicine and Pain Medicine, University Hospital of Homburg/Saar, Kirrberger Strasse 100, 66421 Homburg, Germany; 4 Department of Anaesthesiology and Intensive Care Medicine, University of Luebeck, Ratzeburger Allee 160, 23538 Luebeck, Germany; 5 Department of Anaesthesia, Intensive Care, Emergency and Pain Medicine, Hospital Mechernich, St.-Elisabeth-Strasse 2-6, 53894 Mechernich, Germany; 6 Department of Anaesthesiology, University of Vienna, Währinger Gürtel 18-20, 1090 Vienna, Austria; 7 Department of Intensive Care Medicine, University of Zurich, Rämistrasse 100, 8091 Zurich, Switzerland; 8 Department of Anaesthesiology and Intensive Care Medicine, University of Frankfurt, Theodor-Stern-Kai 7, 60590 Frankfurt, Germany; 9 Department of Clinical Medicine, Saarland University, Campus Homburg, Kirrberger Strasse 100, 66421 Homburg, Germany; 10 Institute for Medical Biometry, Epidemiology and Medical Informatics, Saarland University, Campus Homburg, Kirrberger Strasse 100, 66421 Homburg, Germany

## Erratum to: Ann. Intensive Care (2016) 6:49 DOI 10.1186/s13613-016-0148-2

The original version of this article [1] should have included a list of the collaborators as part of the ICU-CardioMan Investigators group in the acknowledgements list.

The updated version of the acknowledgements is present below.
