# Supplementary material for: Practice of hemodynamic monitoring and management in German, Austrian, and Swiss intensive care units: the multicenter cross-sectional ICU-CardioMan Study
Source: Ann Intensive Care. 2016 May 31;6:49. doi: 10.1186/s13613-016-0148-2 (PMC4887453; doi:10.1186/s13613-016-0148-2)
Supplement: Supplementary file 1 — Additional file 1: Table a1. Available monitoring modalities stratified according to hospital size and academic affiliation. [file 13613_2016_148_MOESM1_ESM.docx]

Table a1. Available monitoring modalities stratified according to hospital size and academic affiliation

|  |  | ≤ 500 beds  n = 25 | 501-1000 beds  n = 48 | >1000 beds  n = 87 |  | Non- University Hospital  n = 63 | University Hospital  n = 97 |
| --- | --- | --- | --- | --- | --- | --- | --- |
| **Invasive pressure Monitoring** | | | | | | | |
| Invasive Pressure Monitoring |  | 100% | 100% | 100% |  | 100% | 100% |
| Automated PPV |  | 28% | 45.8% | 37.9% |  | 38.1% | 39.2% |
|  |  |  |  |  |  |  |  |
| **Non-and minimally invasive extended monitoring** | | | | | | | |
| Plethysmography,  volume clamp |  | 4% | 10.4% | 6.9% |  | 9.5% | 6.2% |
| Bioimpedance |  | 0% | 4.2% | 0% |  | 3.2% | 0% |
| Oesophageal Doppler |  | 28% | 11.6% | 5.7% |  | 12.7 % | 9.3 % |
|  |  |  |  |  |  |  |  |
| **Echocardiography** | | | | | | | |
| Transthoracic |  | 96% | 91.7% | 96.6% |  | 96.8% | 93.8% |
| Transoesophageal |  | 88% | 85.4% | 85.1% |  | 90.5% | 82.5% |
|  |  |  |  |  |  |  |  |
| **Semi-invasive extended monitoring** | | | | | | | |
| Autocalibrated pulse contour analysis |  | 25% | 50% | 40.2% |  | 30.2% | 47.9% |
| Continuous Central venous oxygen saturation (ScvO_2_) |  | 16.7% | 33.3% | 33.3% |  | 22.2% | 36.5% |
|  |  |  |  |  |  |  |  |
| **Invasive extended hemodynamic monitoring** | | | | | | | |
| Transpulmonary lithium dilution |  | 4.2% | 2.1% | 5.7% |  | 3.2% | 5.2% |
| Transpulmonary thermodilution |  | 91.7% | 93.8% | 86.2% |  | 93.7% | 86.5% |
| Pulmonary artery thermodilution |  | 45.8% | 85.4% | 78.2% |  | 69.8% | 79.2% |
