# Supplementary figures and images for: Practice of hemodynamic monitoring and management in German, Austrian, and Swiss intensive care units: the multicenter cross-sectional ICU-CardioMan Study
Source: Ann Intensive Care. 2016 May 31;6:49. doi: 10.1186/s13613-016-0148-2 (PMC4887453; doi:10.1186/s13613-016-0148-2)

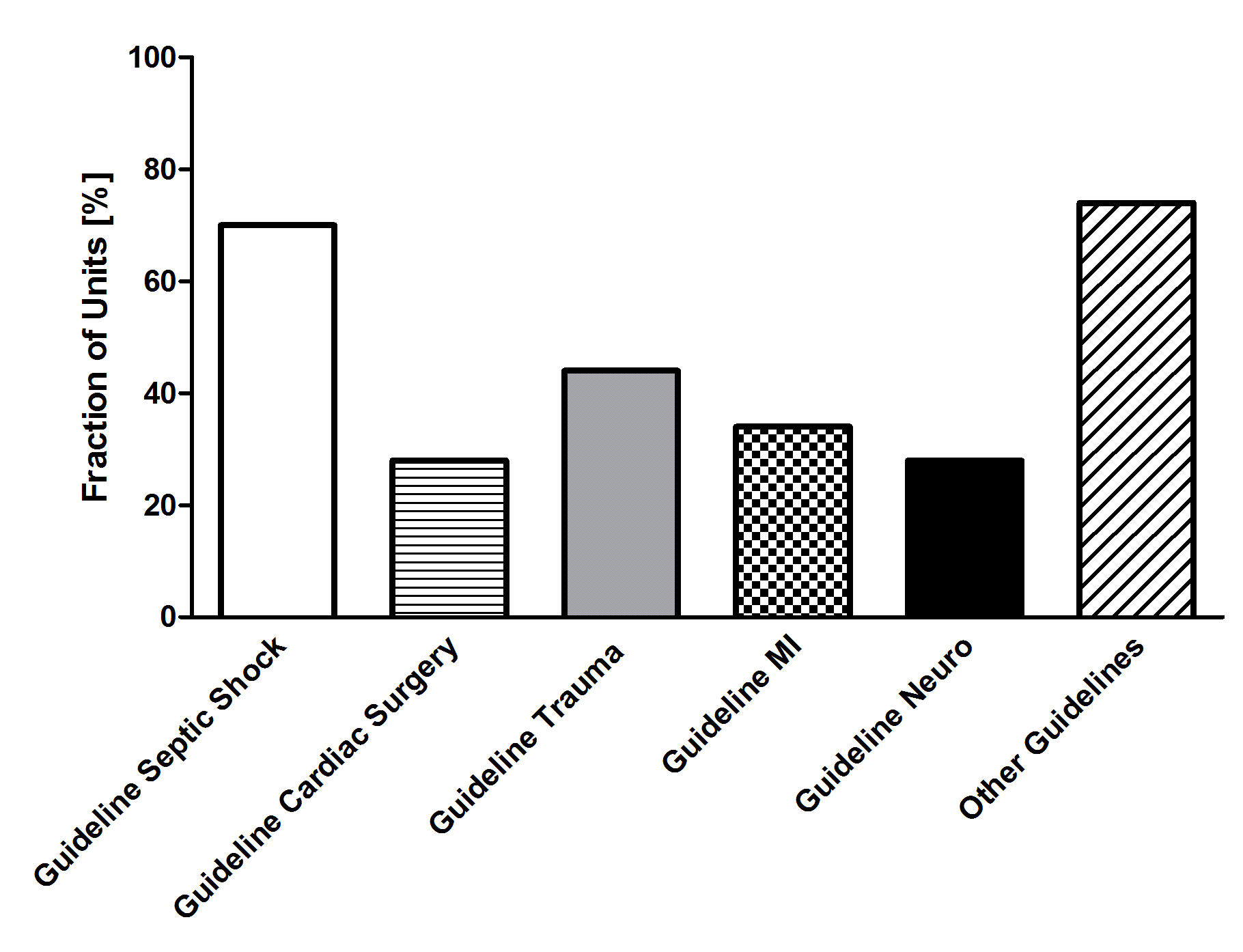

Supplement: Supplementary file 2 — Additional file 2: Figure a1. Implemented treatment protocols Figure a1 illustrates, in how many percent of units the different treatment protocols, that are relevant for hemodynamic management, were implemented (MI = myocardial infarction). [file 13613_2016_148_MOESM2_ESM.bmp]
