# Supplementary material for: Practice of hemodynamic monitoring and management in German, Austrian, and Swiss intensive care units: the multicenter cross-sectional ICU-CardioMan Study
Source: Ann Intensive Care. 2016 May 31;6:49. doi: 10.1186/s13613-016-0148-2 (PMC4887453; doi:10.1186/s13613-016-0148-2)
Supplement: Supplementary file 3 — Additional file 3: Table a2. Implemented haemodynamic treatment protocols stratified to size of hospital and academic affiliation. [file 13613_2016_148_MOESM3_ESM.docx]

Table a2. Implemented haemodynamic treatment protocols stratified to size of hospital and academic affiliation

|  | ≤ 500 beds  n = 25 | 501-1000 beds  n = 48 | > 1000 beds  n = 87 |  | Non- University  n = 63 | University  n = 97 |
| --- | --- | --- | --- | --- | --- | --- |
| Septic shock | 64% | 81.3% | 65.5% |  | 65.1 % | 73.2% |
| Cardiac surgery | 16% | 18.8% | 36.8% |  | 7.9% | 41.2% |
| Neurosurgery | 0% | 31.3% | 34.5% |  | 15.9% | 36.1% |
| Trauma | 32% | 50% | 43.7% |  | 38.1% | 47.4 % |
| Myocardial infarction | 44% | 39.6% | 28.7% |  | 42.9 % | 28.9 % |
| Others | 16% | 25% | 12.6% |  | 15.9% | 17.5% |
