# Supplementary material for: Practice of hemodynamic monitoring and management in German, Austrian, and Swiss intensive care units: the multicenter cross-sectional ICU-CardioMan Study
Source: Ann Intensive Care. 2016 May 31;6:49. doi: 10.1186/s13613-016-0148-2 (PMC4887453; doi:10.1186/s13613-016-0148-2)
Supplement: Supplementary file 4 — Additional file 4: Table a3. Characteristics of included patients. [file 13613_2016_148_MOESM4_ESM.docx]

Table a3. Characteristics of included patients

|  |  |  | n (%) |
| --- | --- | --- | --- |
| Reasons for admission | Post-surgery |  | 895 (50.0%) |
|  |  | Scheduled | 686 (38.3%) |
|  |  | Unscheduled | 209 (11.7%) |
|  | Emergency |  | 819 (45.8%) |
|  |  | Cardiac | 176 (9.8%) |
|  |  | Respiratory | 189 (10.6%) |
|  |  | Trauma | 135 (7.5%) |
|  |  | Sepsis | 116 (6.5%) |
|  |  | Others | 308 (17.2%) |
|  | Others |  | 75 (4.2%) |
| Leading medical discipline | Anaesthesia |  | 511 (28.6%) |
|  | Surgery |  | 226 (12.6%) |
|  | Cardiac Surgery |  | 354 (19.8%) |
|  | Medical |  | 241 (13.5%) |
|  | Interdisciplinary |  | 109 (6.1%) |
|  | Neurosurgical |  | 136 (7.6%) |
|  | Neurological |  | 56 (3.1%) |
|  | Trauma |  | 66 (3.7%) |
|  | Others |  | 89 (5.0%) |
| Type of ward | ICU |  | 1544 (86.3%) |
|  | IMC |  | 245 (13.7%) |
| Severity of illness | SAPS |  | 36.8 ± 18.9 |
|  | TISS |  | 20.2 ± 17.9 |
| Mechanical ventilation | Invasive |  | 775 (43.3%) |
|  | Non-invasive |  | 99 (5.5%) |
|  |  | Controlled | 409 (22.9%) |
|  |  | Assisted | 477 (26.7%) |
|  | None |  | 915 (51.1%) |
| Cardiovascular conditions | Cardiac rhythm |  |  |
|  |  | Sinus | 1366 (76.4%) |
|  |  | Pacer | 130 (7.3%) |
|  |  | AF | 280 (15.7%) |
|  |  | Others | 13 (0.7%) |
|  | Catecholamines/Vasopressors |  | 702 (39.2%) |
| Treatment by guidelines |  |  | 1037 (58%) |

ICU = Intensive care unit; IMC = Intermediate care unit; SAPS = Simplified acute physiology score (severity of illness); TISS = Therapeutic intervention scoring system (severity of illness); AF = Atrial fibrillation.
