# Supplementary material for: Practice of hemodynamic monitoring and management in German, Austrian, and Swiss intensive care units: the multicenter cross-sectional ICU-CardioMan Study
Source: Ann Intensive Care. 2016 May 31;6:49. doi: 10.1186/s13613-016-0148-2 (PMC4887453; doi:10.1186/s13613-016-0148-2)
Supplement: Supplementary file 5 — Additional file 5: Table a4. List of Co-Investigators. [file 13613_2016_148_MOESM5_ESM.docx]

Table a4. List of Co-Investigators

We furthermore want to thank all ICU-CardioMan Investigators for their substantial contribution to acquisition of data, as there are:

Gerd Werner Albuszies, Ruesselsheim, Germany

Rudolf Alexi, Bad Arolsen, Germany

Andreas Bauer, Munich, Germany

Stefan Bergt, Rostock, Germany

Ralph Berroth, Ludwigsburg, Germany

Frank Bloos, Jena, Germany

Hendrick Bracht, Ulm, Germany

Patrick Brass, Krefeld, Germany

Dirk Breukelmann, Hagen, Germany

Andreas Brunauer, Salzburg, Austria

Matthias Burghardt, Dresden, Germany

Michael Christ, Nuernberg, Germany

Beatrix Dietrich, Augsburg, Germany

Christian Diwok, Rostock, Germany

Christian Dohmen, Cologne, Germany

Michael Dueck, Eschweiler, Germany

Ingolf Eichler, Dortmund, Germany

Joerg Engel, Wetzlar, Germany

Regula Erb, Baden, Switzerland

Florian Eyer, Munich, Germany

Andreas Faltlhauser, Weiden in der Oberpfalz, Germany

Florian Feld, Aachen, Germany

Ann Kristin Fricke, Oldenburg, Germany

Bernd Fröhmert, Hamburg, Germany

Wolfgang Funk, Amberg, Germany

Harald Genzwuerker, Buchen, Germany

Hans-Ulrich Giesen, Solingen, Germany

Hans-Hinrich Grafelmann, Wittmund, Germany

Joachim Grosse, Wesel, Germany

Marco Gruss, Hanau, Germany

Christoph Gussone, Bochum, Germany

Hendrik Haake, Moenchengladbach, Germany

Markus Heim, Munich, Germany

Anke Heinemann, Moenchengladbach, Germany

Michael Henrich, Giessen, Germany

Holger Herff, Cologne, Germany

Guido Hermanns, Ueberlingen, Germany

Klaus Hofmann-Kiefer, Munich, Germany

Andreas Hohn, Bochum, Germany

Frauke Honig, Hannover, Germany

Tobias Huebner, Uerberlingen, Switzerland

Tobias Hueppe, Luenen, Germany

Nadine Huelpuesch, Lahr/Schwarzwald, Germany

Judith Ibba, Moenchengladbach, Germany

Ricarda Ibscher, Potsdam, Germany

Michael Irlbeck, Munich, Germany

Johannes Kalbhenn, Freiburg, Germany

Barbara Kapfer, Munich, Germany

Ines Kaufmann, Munich, Germany

Thomas Kirschning, Mannheim, Germany

Stephan F. Kloesel, Ruesselsheim, Germany

Karin Kobusch, Hannover, Germany

Matthias Kochanek, Cologne, Germany

Alf Kozian, Magdeburg, Germany

Oliver Krull, Stendal, Germany

Thomas Kuhl, Krefeld, Germany

Simone Lindau, Frankfurt, Germany

Ingo Lorenz, Innsbruck, Austria

Stefan Maisch, Hamburg, Germany

Gernot Marx, Aachen, Germany

Thomas Marx, Munich, Germany

Onnen Moerer, Goettingen, Germany

Benjamin Moser, Cologne, Germany

Ulrike Mueller, Leipzig, Germany

Stefan Nicolas, Krefeld, Germany

Ludwig Ney, Munich, Germany

Andreas Niedeggen Eschweiler, Germany

Stefan Otto, Saarlouis, Germany

Kevin Pilarczyk, Essen, Germany

Matthias Popp, Freital, Germany

Christian Putensen, Bonn, Germany

Ralf Quabach, Solingen, Germany

Maximilian Ragaller, Dresden, Germany

Axel Ramminger, Berlin, Germany

Stefan Rasche, Dresden, Germany

Sebastian Rehberg, Muenster, Germany

Marcus H. T. Reinges, Giessen, Germany

Reimer Riessen, Tuebingen, Germany

Knut-Age Roehrich, Leipzig, Germany

Rainer Roehrig, Giessen, Germany

Jan P. Roesner, Rostock, Germany

Heiner Ruschulte, Hannover, Germany

Samir G. Sakka, Cologne, Germany

Alexander Schellhaass, Heidelberg, Germany

Jens-Christian Schewe, Bonn, Germany

Ingo Schirotzek, Giessen, Germany

Jörn V. Schley, Hamburg, Germany

Alexander Schmitt, Völklingen, Germany

Martin Schott, Hannover, Germany

Patrick Schramm, Mainz, Germany

Stefan Schroeder, Dueren, Germany

Jan-Karl Schuette, Dueren, Germany

Alexander Schuld, Fulda, Germany

Konrad Schwarzkopf, Saarbruecken, Germany

Julia Seiler, Ingolstadt, Germany

Sixten Selleng, Greifswald, Germany

Philipp Simon, Leipzig, Germany

Kornel Skitek, Cottbus, Germany

Jens Soukup, Cottbus, Germany

Markus Stephan, Stuttgart, Germany

Henning Stetefeld, Cologne, Germany

Klaudiusz Suchodolski, Hannover, Germany

Stefan Toennies, Marl, Germany

Georg Trummer, Freiburg, Germany

Michael Wattenberg, Bremen, Germany

Stefan P. Wirtz, Bad Saarow, Germany

Hermann Wrigge, Leipzig, Germany

Christian Wunder, Wuerzburg, Germany

Cagatay Yildirim, Marburg, Germany

Kai Zacharowski, Frankfurt aM, Germany

Alexander Zarbock, Muenster, Germany

York Zausig, Regensburg, Germany

Michael Zoller, Munich, Germany
